# New human H5N1 case: Should we worry? A genetic perspective

**DOI:** 10.1016/j.nmni.2024.101510

**Published:** 2024-10-11

**Authors:** Francesco Branda, Massimo Ciccozzi, Fabio Scarpa

**Affiliations:** Unit of Medical Statistics and Molecular Epidemiology, University Campus Bio-Medico of Rome, Rome, Italy; Department of Biomedical Sciences, University of Sassari, Sassari, Italy

**Keywords:** Genetic variability, Phylogenetic analysis, Spillover event, Human-to-human transmission, One health surveillance

Dear Editor,

On September 6, 2024, the CDC confirmed a new human case of avian influenza A(H5) reported by the state of Missouri (https://www.cdc.gov/media/releases/2024/s0906-birdflu-case-missouri.html). This case represents the 14th human case of H5 in the United States in 2024, and the first where the individual had no known occupational exposure to sick or infected animals (see Fig. 1).

**Fig. 1:**
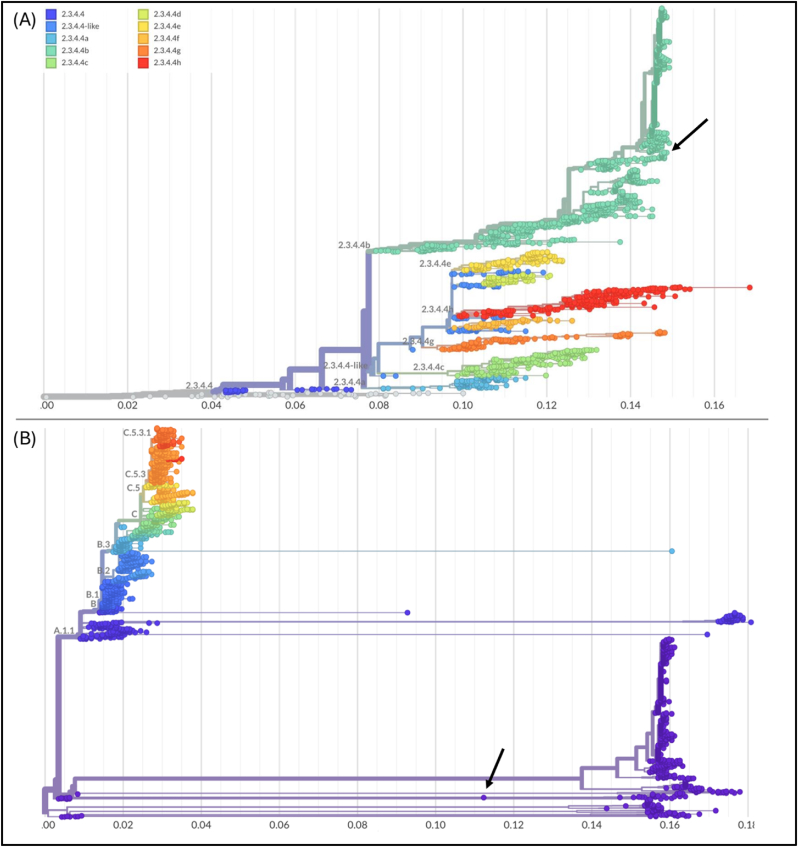
(A) Phylogenetic reconstructions of the H5N1 subtype based on all available HA sequences from GISAID (https://gisaid.org). The legend indicates the clade of each terminal. (B) Phylogenetic reconstruction based on the NA gene, including all available sequences of the type N1. The arrows highlight the sequence of the human case from Missouri (EPI_ISL_19413343). The scale in the x-asses is espressed in genetic distance. Analyses were performed using the Nextstrain/ncov tool available at the GitHub repository (https://github.com/nextstrain/ncov). The figure was edited using GIMP 2.8 (https://www.gimp.org/downloads/oldstable/).

As is often the case in similar situations, concerns arose about a potential reassortment leading to a true species jump, especially since the neuraminidase molecule had not been initially typed and the patient does not appear to have had contact with infected or diseased animals (https://edition.cnn.com/2024/09/13/health/missouri-bird-flu-h5n1/index.html). The genetic analysis proposed here confirms that the new case belongs to the H5N1 subtype, clade 2.3.4.4b, which is the same as the previous cases from the USA [1]. Phylogenetic reconstructions of both HA and NA genes indicate that the genetic variability of the specimen from the new human case in Missouri (EPI_ISL_19413343) falls within the range of well-known strains. The genetic variability of HA remains low, showing limited variability over time, with a distance of 0.1 from the root and seven amino acid substitutions along with 56 reversions to the root, which is temporally placed around 2014. The genetic variability of NA is similarly limited, with minor fluctuations over time and a divergence of 0.1 from the root. Compared to HA, NA evolves slightly faster, with 45 amino acid substitutions, two reversions, and one gap relative to the root. In both cases, the nucleotide and amino acid changes do not appear to have introduced enough genetic variability to significantly diverge from other samples of the same subtype and clade. The phylogenetic pattern observed does not suggest a typical spillover event but rather an incidental host occurrence. Indeed, the spillover is an event that can be clearly identified at a genetic level once it has occurred. In cases of viral adaptation to a new species, where the virus gains the ability for human-to-human transmission, distinct groups typically form. These are represented by genetic clusters composed of lineages sharing the same genetic traits that confer the new transmission characteristics. Although there have been no reports of H5 outbreaks in cattle in Missouri, H5 outbreaks have been documented in both commercial poultry farms and backyard flocks in 2024. Additionally, H5N1 bird flu has previously been detected in wild birds within the state. To corroborate the scenario depicted by genetic analyses, it should be noted that although there have been no reports of H5 outbreaks in cattle in Missouri, H5 outbreaks have been documented in both commercial poultry farms and backyard flocks in 2024. Additionally, H5N1 bird flu has previously been detected in wild birds within the state (https://www.cdc.gov/media/releases/2024/s0906-birdflu-case-missouri.html). In any case, this analysis does not imply that we should relax our vigilance in monitoring potential threats. Spillover and reassortment are events that can occur, and the only way to stay prepared is to maintain thorough monitoring [2] by sequencing and typing all new cases, both human and non-human. Indeed, on the insights gained from the COVID-19 pandemic, the scientific community acknowledges that continuous One Health surveillance, which includes ongoing genome-based monitoring, is the most effective approach for improving understanding.

## Funding

None.

## CRediT authorship contribution statement

**Francesco Branda:** Conceptualization, Data curation, Investigation, Writing – original draft, Writing – review & editing. **Massimo Ciccozzi:** Conceptualization, Supervision, Validation, Writing – original draft, Writing – review & editing. **Fabio Scarpa:** Conceptualization, Data curation, Formal analysis, Investigation, Visualization, Writing – original draft, Writing – review & editing.

## Declaration of competing interest

The authors declare that they have no known competing financial interests or personal relationships that could have appeared to influence the work reported in this paper.

## References

[1] Branda F., Ciccozzi A., Romano C., Casu M., Sanna D., Ceccarelli G., Ciccozzi M., Scarpa F. (2024). Insights into avian influenza A(H5N1) events: epidemiological patterns and genetic analysis. Infect Diseas.

[2] Branda F., Ciccozzi M., Scarpa F. (2024). H5N1 avian influenza: tracking outbreaks with real-time epidemiological data. Lancet Infect Dis.

